# A rare *BCR-ABL1* transcript in Philadelphia-positive acute myeloid leukemia: case report and literature review

**DOI:** 10.1186/s12885-019-5265-5

**Published:** 2019-01-10

**Authors:** Monica Piedimonte, Tiziana Ottone, Valentina Alfonso, Antonella Ferrari, Esmeralda Conte, Mariadomenica Divona, Maria Paola Bianchi, Maria Rosaria Ricciardi, Simone Mirabilii, Roberto Licchetta, Alessia Campagna, Laura Cicconi, Giulia Galassi, Sabrina Pelliccia, Annapaola Leporace, Francesco Lo Coco, Agostino Tafuri

**Affiliations:** 1 Department of Clinical and Molecular Medicine, Hematology Sant’Andrea University Hospital, Sapienza University of Rome, Rome, Italy; 2 Department of Biomedicine and Prevention, University of Tor Vergata, Rome, Italy

**Keywords:** Acute myeloid leukemia, Philadelphia chromosome, BCR-ABL1 e6a2, Atypical transcripts, TKI

## Abstract

**Background:**

Philadelphia (Ph) chromosome results from the reciprocal translocation t(9;22)(q34.1;q11.2) and is diagnostic for chronic myeloid leukemia (CML). However, this translocation is also found in acute lymphoid leukemia (ALL), as well as in rare cases of acute myeloid leukemias (AML). Most patients with CML harbor either the e13a2 or the e14a2 BCR-ABL fusion product, while a small subset of the cases expresses e1a2 or e19a2 transcripts. Moreover, several atypical *BCR-ABL1* transcripts, beside the most common e1a2, e13a2 and e14a2, have been described, mainly in patients with CML. However, ALL and de novo AML may also carry *BCR-ABL1* atypical transcripts which will confer a poor prognosis.

**Case presentation:**

A 78-years old male was admitted at our hospital with clinical and laboratory features allowing to make the diagnosis of AML. No evidence of a preceding CML (splenomegaly or basophilia) was found. The karyotype on G-banded metaphases was 46,XY, t(9;22)(q34;q11). While the molecular analysis was ongoing, the patient started treatment based on hydroxyurea followed by 5-aza-2′-deoxycytidine. The molecular biology analysis revealed the simultaneous presence of the common p190 e1a2 and the rare e6a2 isoforms. Because of persistent pancytopenia and presence of blasts, according to the molecular data, he was then switched to tyrosine kinase inhibitors (TKIs) treatment. Nevertheless, after 2 months, the patient was still refractory to second line treatment dying because of a pulmonary infection.

**Conclusion:**

The atypical p190 e6a2 transcript seems to be associated in AML with aggressive disease. TKI therapy alone does not seem to control the disease. Prompt observations on these patients carrying rare *BCR-ABL1* transcripts may help to establish optimal treatment approaches on these aggressive BCR-ABL1 phenotypes in different setting of patients.

## Background

The t(9;22) chromosome translocation originating the BCR-ABL1 fusion oncoprotein is detected in more than 95% of patients with CML representing the hallmark of the disease [1]. However, this translocation is also found in 10 to 20% of adults and in 2 to 5% of children with ALL, as well as in rare cases (1% approximately) of AML [2].

The breakpoint on chromosome 9 is localized between exons 1a, 1b and a2. In contrast, breakpoints on chromosome 22 occur within the *BCR* gene, consisting of 23 exons, thus resulting in several distinct fusion genes. Generally, the breakpoints location on *BCR* gene occur between b1-b5 exons, within a central region called major breakpoint cluster region (M-bcr), whereas rare cases occur within two other breakpoint cluster regions: minor (m-bcr), with the breakpoint between e1-e2 exons, and micro (μ-bcr) with rupture point between e19-e20 exons. Depending on the breakpoint of the *ABL1* gene, the fusion protein has different molecular weight: p190, p210 and p230 proteins for m-bcr, M-bcr and μ-bcr, respectively [3]. Moreover, atypical *BCR* breakpoints outside the cluster regions have been described, involving splicing between whole exons, insertion of small sequences or genomic breakpoints within exons. For example, e8a2, e15a2 and e6a2 have been also described and their clinical significance is under investigation [2, 4, 5]. The atypical e6a2 *BCR-ABL1* transcript produces a rare fusion protein of 185 kDa conferring a poor prognosis in CML due to its association with aggressive phenotype and early transformation, perhaps due to the lack of an important regulatory BCR sequence within the fusion proteins [6]. Here we report a rare case of de novo AML carrying the *BCR-ABL1* transcript e6a2.

## Case presentation

A 78-years old male was admitted to the Hematology of the University Hospital Sant’Andrea-Sapienza, because of worsening fatigue and abdominal pain. Written informed consent was obtained from the patient and the study was approved by our institutional review board.

The peripheral blood count showed hyperleucocytosis (WBC 118 × 10^9^/L), anemia (hemoglobin 8.6 g/dl) and mild thrombocytopenia (98 × 10^9^/L), with no associated splenomegaly. Peripheral blood smear showed hypercellularity with 90% blast cells.

**Fig. 1:**
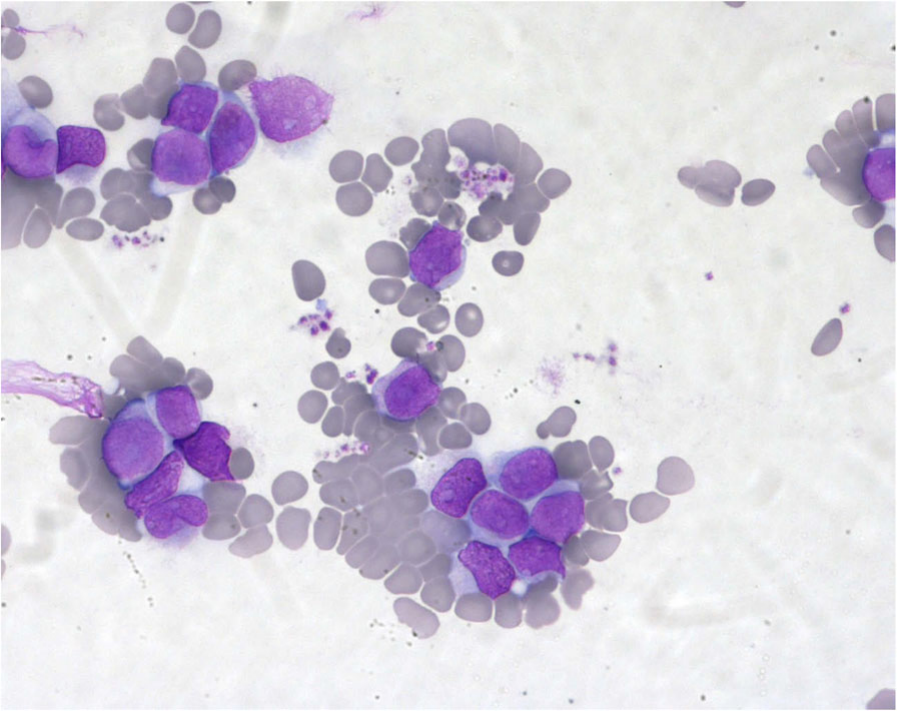
The bone marrow cell morphology of the patient at diagnosis. Agranular blast cells of medium and large size

The morphological examination of bone marrow (BM) aspirate showed 90% agranular blast cells of medium and large size (Fig. 1) and the immunophenotypic analysis performed on a FACScalibur flow cytometer using standard protocol revealed that blast cells were CD34+, CD117+, CD33+, CD13+, HLA-DR+, CD2+ MPO+/−, CD7+/− [7].

A diagnosis of AML (M2) was established and the patient started cytoreduction with hydroxyurea obtaining after seven days of treatment a WBC count of 39 × 10^9^/L.

Conventional karyotyping was performed on the BM diagnostic aspirate after short-term culture and analyzed after G-banding. The description of the karyotype was made according to the International System for Human Cytogenetic Nomenclature. The cytogenetic analysis on G-banded metaphases disclosed a 46,XY,t(9;22)(q34;q11) karyotype. Then, interphase FISH experiments were carried out using BCR-ABL1 probes (Vysis) and demonstrated the presence of *BCR-ABL1* fusion gene.

**Fig. 2:**
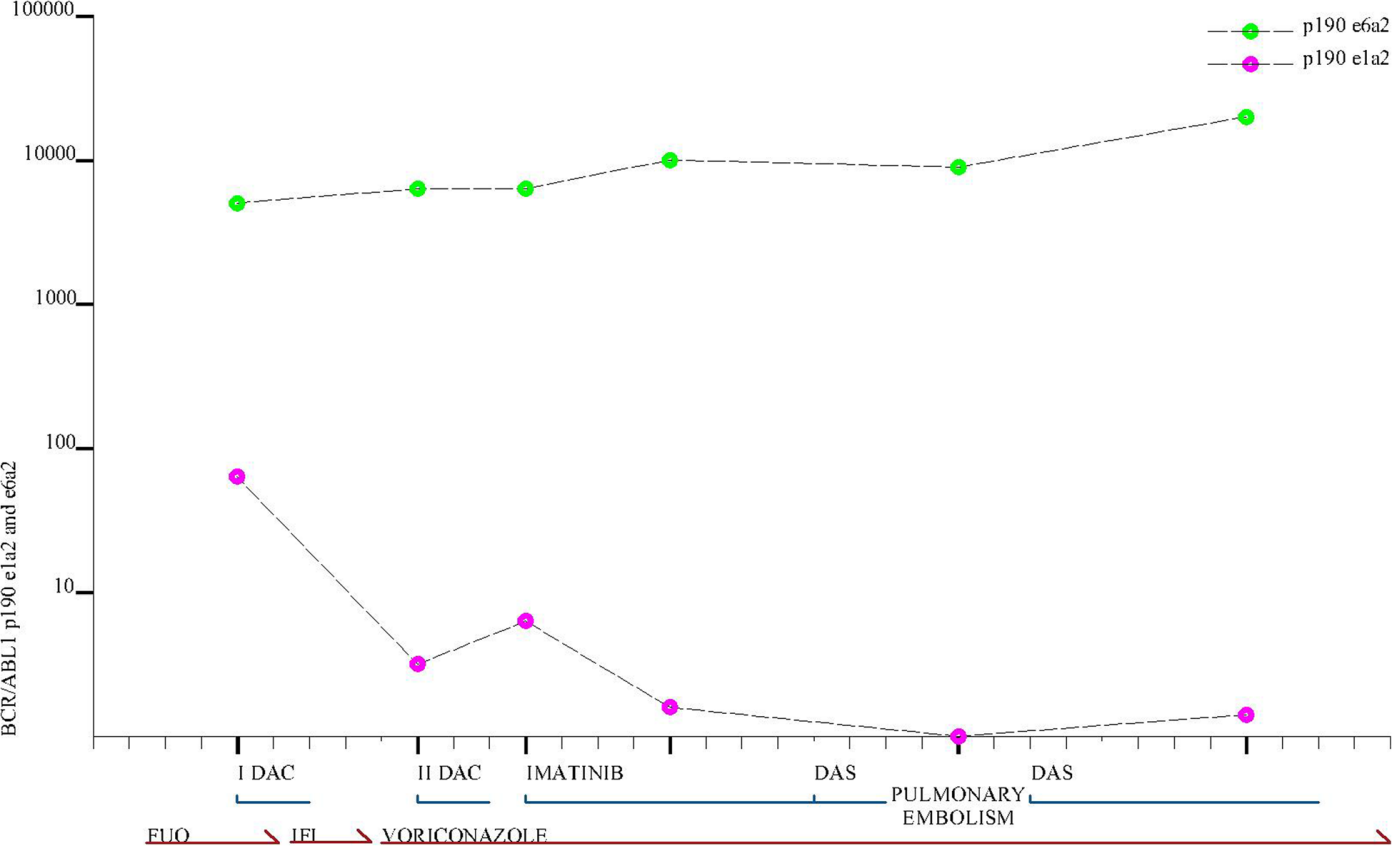
Kinetics of BCR-ABL1 p190 e1a2 and e6a2 at diagnosis and during follow-up and treatment course of the AML patient

At the resolution of a pulmonary aspergillus infection treated with voriconazole, while the cytogenetic and molecular analyses were ongoing, the patient started treatment with 5-aza-2′-deoxycytidine (otherwise called decitabine, 20 mg/m^2^ for 5 days) for a total of two cycles. Subsequently, nested RT-PCR revealed the simultaneous presence of the common p190 e1a2 and the rare e6a2 isoforms (Fig. 2). PCR products, corresponding to BCR-ABL1 p190 amplification, were purified from agarose gel (QIAquick PCR Purification Kit - Qiagen) and directly sequenced on ABI/Prism 3130 Sequencer (Thermo Fisher Scientific) using BigDye® Terminator v3.1 Cycle Sequencing Kit (Thermo Fisher Scientific). RQ-PCR for e6a2 *BCR-ABL1* transcript analysis was performed using the forward primer Q-BCR_ex6_F (GATCCAACGACCAAGAACTCTCT), the reverse primer ENR561 and the ENP541 probe reported elsewhere [8]. The p190 e6a2 values were normalized on the number of *ABL1* transcripts and expressed as the number of p190 e6a2 copies every 10^4^ copies of *ABL1*. To assess molecular response after treatment, a real-time quantitative RT-PCR assay (RQ-PCR) for p190 e6a2 was designed by which we observed significant different kinetics through the e1a2 and e6a2 transcripts with consistent persistence of e6a2 [9].

After the first, BM aspirated showed 70% blast cells and two transcripts e1a2 and e6a2 were respectively 3.09 and 5805.47/10^4^ ABL1, while after second cycles blast cells were 20% and e1a2 and e6a2 5.71 and 5747.52.

Because of persistent pancytopenia and presence of blasts after two cycles of decitabine and in light of molecular data, the patient was then switched to TKI treatment.

The initial therapy consisted of imatinib 600 mg/day for two weeks that was subsequently reduced to 400 mg/day due to febrile neutropenia. After one month of imatinib, bone marrow showed 60% blast cells with small improvement of thrombocytopenia. Therefore, treatment was switched to dasatinib 100 mg/day, but it was discontinued five days later because of pulmonary embolism. At 10 days of TKI discontinuation, e1a2 and e6a2 were 0.17 and 9477.16/10^4^ ABL1, respectively. After two months of continuous therapy with TKIs, bone marrow infiltration was present and the two transcripts were e1a2 1.6 and e6a2 23727.06/10^4^ ABL1 (Fig. 2).

The patient, still refractory to second-line treatment, died of pulmonary infection.

## Discussion

The AML with *BCR-ABL1* fusion is a rare entity and has been included in the 2016 revised World Health Organization (WHO) as a provisional entity of myeloid neoplasm and acute leukemia [10]. The 2017 European LeukemiaNet risk stratification allocated AML with BCR-ABL1 to the adverse risk group [11]. In fact, the patients with BCR-ABL1 positive AML are often refractory to chemotherapy while the responses to imatinib are of limited duration [12]. A recent paper has reported that allogenic stem cell transplantation (allo-SCT) may improve outcome of younger patients [13]; however, given the small number of cases, the optimal therapy for AML with BCR-ABL1 has not been established yet. Beside common BCR-ABL1 transcripts, unusual transcripts have been also described in CML and acute leukemias. We report a rare case of AML characterized by the co-expression of the atypical e6a2 BCR-ABL1 transcript with the common e1a2 one. The e6a2 transcript is mainly observed in CML and sporadically in acute leukemia. In particular, so far about 24 e6a2 cases, in addition to our, have been described in the literature: 17 in CML (1 developed after chronic myelomonocytic leukemia -CMML-), 1 in a case of acute basophilic leukemia, 5 in AML (3 de novo and 2 cases developed after CMML and Myelofibrosis respectively) and 1 in an ALL patient [14]. Table 1 is a summary of the clinical features of e6a2 BCRABL-positive leukemia patients described so far. Notably, co-expression of e6a2 and e1a2 transcripts was previously described in only few cases of CML [15, 16]. Here we reported the first evidence of coexpression in AML.

**Table 1:** Cases reported in literature showing the e6a2 transcript

Diagnosis	Sex/age	Coexpression of other transcripts	Other molecular alterations	Treatment	References
CP - CML	M/41			BMT	Hochhaus et al. [2]
CP - CML	M/50			IFN-Ara C-- > BMT	Dupont et al. [30]
BC - CML	M/65			Imatinib	Schultheis et al. [6]
CP - CML	M/76			IFN	Colla et al. [31]
CML secondary to CMML	F/64		t(11;16)	Imatinib	Hayette et al. [32]
CP - CML	M/37	e1a2		Imatinib	Roti et al. [16]
Acute basophilic leukemia	M/71			Imatinib-Dauno-Ara C	Gregoire et al. [33]
ALL	F/29			G MALL 7/03-Imatinib-- > BMT	Burmeister et al. [34]
CP - CML	M/43			Imatinib	Breccia et al. [35]
AML	F/53			Imatinib-Ida-Ara C -- > BMT-Dasatinib	Ritchie et al. [22]
AML (M7)	F/53		del(18)(p10)	Imatinib-Ida-Ara C -- > Dasatinib-BMT	Corm et al. [23]
CP - CML	M/48			Imatinib-- > Dasatinib	Schnittger et al. [36]
CP - CML	M/48		t(7;9), ins(22;9)	Imatinib-- > BMT	Vefring et al. [17]
AP - CML	M/42			Imatinib -- > BMT-Dasatinib	Vefring et al. [17]
CP - CML	M/67			Imatinib	Popovici et al. [26]
CP - CML	F/18			Imatinib	Torres et al. [37]
AP - CML	M/57		trisomy 8	Imatinib	Beel et al. [18]
CP - CML	M/36	e1a2		Imatinib-Nilotinib -- > BMT	Langabeer et al. [15]
AP - CML	M/51			Imatinib-- > BMT	Rohon et al. [19]
BC – CML	M/48			Dasatinib	Zagaria et al. [14]
AML (M4)	F/55			Mitox-Ara C-Imatinib -- > BMT-Dasatinib-DLI	Harada et al. [21]
BC - CML	F/48		trisomy and tetrasomy 8	Dauno-Ara c-Imatinib -- > BMT	Crampe et al. [20]
AML secondary to CMML	M/58		DNMT3A RUNX1 SUZ12	3 + 7-Dasatinib-Nilotinib	Yao et al. [25]
AML secondary to myelofibrosis	M/80		JAK2-V617F	Hydroxyurea-valproic acid Dasatinib-Imatinib	Brattas et al. [38]

Data reported so far indicate that the course of disease associated with e6a2a is particularly aggressive both in chronic and acute leukemias. In fact, within CML cases harboring this transcript, 6/17 developed an accelerated or blastic phase [6, 14, 17–20]. Furthermore, there was a prevalence of males on female (ratio 14:3) and the median age at diagnosis was 48 years old (range 18–76 years). Notably, the 3 de novo AML cases with e6a2 transcript were female and the age at diagnosis ranged from 53 to 55 years old [21–23]. These patients underwent allo-SCT in first CR after TKIs and chemotherapy. In these cases, dasatinib and nilotinib were effective treatments in inducing molecular remission after imatinib failure [22, 23] and against relapse after transplant maybe thanks to synergy between TKI and the graft versus-leukemia effect, reaching sustained response [21, 22]. They were alive at 24, 30 and 52 months from diagnosis [21–23]. On the contrary, the case here presented was older and not eligible to allo-SCT. The absence of prior clinical or laboratory evidences of CML (splenomegaly or basophilia) suggested a diagnosis of de novo AML with *BCR-ABL1* [24]. He was resistant to decitabine and to TKIs and he died for disease shortly after the diagnosis.

Despite the clinical heterogeneity, all previously reported cases and the one here reported indicate that the atypical e6a2 transcript is associated with aggressive disease and underline the potential advantage of TKIs and allo-SCT in improving prognosis of these patients [6, 14, 15, 17–20, 25]. As suggested, the poor prognosis could be attributed to the partial loss of the Guanine Exchange Factor (GEF)/dbl-like domain [26]. This GEF/dbl-like domain is able to mediate the interaction with several Ras-like G proteins involved in proliferation, signalling and cell organization. Therefore, this truncation seems to determine an increased kinase activity with a greater transforming oncogenic potential. In our patient, the significant different kinetics of e1a2 and e6a2 transcripts quantified by RQ-PCR during the treatment further supports this hypothesis.

The case here described presents a diagnostic and therapeutic challenge since it shows the importance of combining conventional karyotype/FISH and appropriate genetic assays to detect rare *BCR-ABL1* fusion transcripts. Furthermore, the different clearance of the e1a2 and e6a2 transcripts underlines the importance to design specific molecular assays for the evaluation of minimal residual disease and to choose the best therapeutic options.

## Conclusions

In conclusion, hematological malignancies with the atypical e6a2 show an aggressive phenotype with an higher capacity of transformation to blast crisis in CML. Ph + AML is more rare, but with the same worse prognosis [15]. Therapeutic options include TKIs and allo-SCT. In fact, these patients can benefit from use of TKI, although this treatment seems to be more effective as salvage or maintenance therapy or even as bridging to transplant after a remission [15].

For patients not eligible to transplantation, the prognosis is even more unfavourable and a different approach should be considered. There is no standard of care and treatment of patients affected by Ph + AML, especially elderly, remains an area of unmet need. Notably, Shao et al. recently reported that dasatinib single agent may be an effective and tolerable induction therapy for AML patients with *BCR-ABL1* and poor physical condition [27]. Hovewer, prognosis with low-intensity therapies is still poor and novel therapeutic strategies are needed. Future approaches may investigate role of Ras-Mek inhibitors, nowadays under investigation as targeted therapies in other diseases. Additional strategies can combine also novel agent: gentuzumab ozogamicin, tipifarnib, volasertib [28]. Moreover, interesting data arise from the use of ibrutinib [29] that inhibits AML blast proliferation, enhancing cytotoxic activities of cytarabine or daunorubicin.

Prompt observations of patients carrying rare *BCR-ABL1* transcripts associated with unfavourable prognosis remain mandatory to include these patients in more aggressive treatment strategies. In vitro studies are necessary to clarify the transforming property and the mechanisms of resistance of e6a2 transcript and to design novel target therapeutic agents.

## Abbreviations

ALL: Acute lymphoid leukemia
AML: Acute myeloid leukemia
BM: Bone marrow
BMT: Bone marrow transplantation
CM: Chronic myeloid leukemia
DAC: Dacogen
DAS: Dasatinib
FUO: Fever of unknown origin
GEF: Guanine exchange factor
IFI: Invasive fungal disease
M-bcr: Major breakpoint cluster region
m-bcr: Micro breakpoint cluster region
m-bcr: minor breakpoint cluster region
MRD: Minimal residual disease
Ph: Philadelphia
RQ-PCR: Real-time quantitative RT-PCR
TKIs: Tyrosine kinase inhibitors
WBC: White blood cells
